# The Extraction and Characterization of Pseudorange Multipath Based on BDS-3 Multi-Frequency Observations

**DOI:** 10.3390/s23136151

**Published:** 2023-07-04

**Authors:** Zhongchen Guo, Xuexiang Yu, Chao Hu, Chuang Jiang, Hao Tan, Mingfei Zhu, Shicheng Xie

**Affiliations:** 1 Key Laboratory of Aviation-Aerospace-Ground Cooperative Monitoring and Early Warning of Coal Mining-Induced Disasters of Anhui Higher Education Institutes, Anhui University of Science and Technology, Huainan 232001, China; 2020100003@aust.edu.cn; 2 School of Environment and Surveying Engineering, Suzhou University, Suzhou 234000, China; 3 School of Earth and Environment, Anhui University of Science and Technology, Huainan 232001, China; 2021100002@aust.edu.cn (M.Z.); xsc123@aust.edu.cn (S.X.); 4 School of Geomatics, Anhui University of Science and Technology, Huainan 232001, China; chaohu2014gnss@163.com (C.H.); jiangch@aust.edu.cn (C.J.); 2021217@aust.edu.cn (H.T.)

**Keywords:** BDS-3, pseudorange multipath, multi-frequency observations, analytical expression, combination noise, ionospheric delay, correlation analysis

## Abstract

Global Navigation Satellite System (GNSS) observations are subject to various errors during their propagation process. A reasonable correction of these errors can improve the positioning, navigation, and timing (PNT) service capability. The impact of multipaths on pseudorange observations can reach a decimeters or even meters level. However, their mechanism is complex and there is currently no universally accepted high-precision correction model. The correlation between the pseudorange multipaths (MP) of BDS-2 satellites and satellite elevation has been confirmed, while there have been fewer analyses of the MP characteristics for different frequencies of BDS-3 satellites. The broadcasting of multi-frequency observations in BDS-3 should theoretically make the extracted MP more accurate compared to traditional methods. Based on this, in this contribution, a multi-frequency MP extraction algorithm based on the least squares principle is proposed, which can simultaneously eliminate the influence of higher-order ionospheric delay. The analytical expression for only eliminating first-order ionospheric delay is successfully derived. Subsequently, the characteristics of the MPs extracted from different frequency combinations and the impact of combination noise on the extraction accuracy are discussed. The influence of second-order ionospheric delay on the MPs for each frequency under different combination noises, as well as the periodic behavior exhibited in long-term observations of the BDS-3 medium earth orbit (MEO) and inclined geosynchronous orbit (IGSO) satellites, are also analyzed. Finally, the correlations between the MPs of each frequency of BDS satellite and elevation are quantitatively analyzed based on observations from 35 stations. Overall, this work has positive implications for the study of the MP characteristics of BDS-3 and subsequent modeling efforts.

## 1. Introduction

GNSS has been widely used in PNT services due to its advantages of high accuracy, high frequency, and all-weather capabilities [1,2]. As the main member of navigation systems, the Beidou Satellite Navigation System (BDS) was independently designed and developed by China and has undergone a “three-step” development strategy. On 31 July 2020, it was officially opened for global service. Compared with other navigation systems, the BDS satellite constellation is composed of three types of orbiting satellites: geostationary orbit (GEO), IGSO, and MEO. Both the IGSO and MEO satellites of BDS-3 can transmit observations of five frequency signals [3,4]. Despite the increased complexity in processing the observations of multi-orbit and multi-frequency signals, this ability significantly improves the overall service performance and accuracy of the system [5,6].

Pseudorange and carrier phase observations are the basic measurements used for positioning in most navigation systems. These measurements are subject to various errors during signal propagation, such as atmospheric delay, ionospheric delay, and multipath effects [7,8,9,10]. Reasonably eliminating or reducing the effects of these errors can effectively improve the positioning accuracy [11,12,13,14]. Previous studies have shown that a reasonable combination of multiple frequency observations can reduce the impact of these errors, such as ionosphere-free combinations, ionosphere-reduced combinations, and geometric-free combinations [15,16,17,18,19,20]. With the increasing number of transmission signals, there are more favorable combinations available.

Due to their complex mechanism and the difficulty in modeling them, it is difficult to accurately correct multipath delay errors during the positioning process. Currently, this issue has been researched by many scholars [21,22,23,24,25]. However, some studies in recent years have shown that BDS is not only affected by the reflected signals from the surrounding environment on the ground, but also contains certain systematic biases in the pseudorange observations on its satellite end. In 2012, Hauschild et al. first discovered the systematic errors of the MPs in BDS-2 MEO satellite signals [26]. Subsequently, relevant scholars analyzed the MPs of three BDS-2 types of satellites and explored model correction algorithms [27,28,29,30]. The results showed that correcting the MP can effectively improve the positioning accuracy. With the gradual launch and deployment of BDS-3 satellites, some scholars have also investigated the signal qualities of various frequencies of BDS-3 and found that the MPs of BDS-3 satellites are significantly smaller than those of BDS-2 [31,32,33]. However, it is worth noting that, when extracting the MP of each BDS-3 satellite frequency, the traditional dual-frequency pseudorange and carrier phase observations combination method is still used and the multi-frequency signals provided by BDS-3 are not fully utilized. In theory, introducing additional carrier phase observations can better extract the true signal of an MP [20,34]. Therefore, how to reasonably use multi-frequency observations to extract the MPs for each frequency should be studied.

The traditional MP extraction method can only obtain a unique solution by eliminating the geometric distance term independent of the frequency and the first-order ionospheric delay term, as it only uses dual-frequency observations. However, when triple-, quad-, or five-frequency observations are used, redundant observations can be obtained, which leads to the optimal solution with the minimum combination noise. Moreover, the introduction of triple-, quad-, and five-frequency observations can make it possible to simultaneously eliminate higher-order ionospheric delay terms. When the first- and second-order ionospheric delay terms are simultaneously eliminated, a unique solution can be obtained using triple-frequency observations, while quad- and five-frequency observations can still achieve optimal solutions. The use of multi-frequency observations can also provide support for analyzing the influence of higher-order ionospheric delay terms on MP extraction.

The method of extracting MPs using BDS-3 multi-frequency observations is mainly discussed in this work, and based on this, the characteristics of the MPs on BDS-3 MEO and IGSO satellites are analyzed. The main structure of the article is arranged as follows. The formula for calculating the optimal combination coefficient of an MP based on multi-frequency observations is derived in Section 2, where a combination coefficient calculation method based on the least squares algorithm is proposed. In Section 3, the characteristics of MPs at various frequencies are studied considering three aspects: the impact of different frequency combinations on MP extraction, the influence of second-order ionospheric delay on MP extraction, and the correlation between MPs and elevation. Finally, some conclusions and the next research plans are given in Section 4.

## 2. Methods

Carrier phase and pseudorange observations are the basic observations broadcasted by each navigation system and their linearized observation equations can be expressed as [35]:

(1) $$\left\{ \begin{array}{l} \Phi_{i}=\rho_{r}^{s}+cdt_{r}-cdt^{s}+T_{r}-k_{i}I_{1,1}-\nu_{i}I_{1,2}+\lambda_{i}N_{i}+\varepsilon_{\Phi_{i}}\\ P_{i}=\rho_{r}^{s}+cdt_{r}-cdt^{s}+T_{r}+k_{i}I_{1,1}+2\nu_{i}I_{1,2}+{\mathrm{MP}}_{i}+\varepsilon_{{}_{P_{i}}} \end{array}\right.$$

where the subscript *i* (*i* = 1, 2,…, *n*) represents different frequencies; Φ and *P* represent the carrier phase and pseudorange observations; $\rho_{r}^{s}$ denotes the geometric distance between the satellite and the receiver; $dt_{r}$ and $dt^{s}$ represent the receiver and satellite clock errors, *c* is the speed of light; *T_r_* represents the tropospheric delay; $k_{i}=f_{1}^{2}/f_{i}^{2}$ and $\nu_{i}=f_{1}^{3}/f_{i}^{3}$ represent the first- and second-order ionospheric delay amplification factors of frequency *i*, respectively, *I*_1,1_ and *I*_1,2_ denote the first- and second-order ionospheric delay of the *f*_1_ frequency, $\lambda_{i}$ and $N_{i}$ represent the wavelength and ambiguity of frequency *i*, MP*_i_* is the pseudorange multipath, and $\varepsilon_{\Phi_{i}}$ and $\varepsilon_{{}_{P_{i}}}$ denote other errors of the carrier phase and pseudorange observations.

Usually, MP*_i_* can be extracted from dual-frequency observations and its expression is [26,31,36]:

(2) $${\mathrm{MP}}_{i}^{}=P_{i}^{}-\frac{f_{i}^{2}+f_{j}^{2}}{f_{i}^{2}-f_{j}^{2}}\cdot \Phi_{i}+\frac{2f_{j}^{2}}{f_{i}^{2}-f_{j}^{2}}\cdot \Phi_{j}-B_{i,j}+\xi$$

where *i* and *j* denote different frequencies, $B_{i,j}$ is the combination of the ambiguity and hardware delay, and ξ represents other errors. When the carrier phase observation does not include cycle slip, $B_{i,j}$ can be treated as a constant, that is, after removing $B_{i,j}$ from the sequence extracted from Equation (2), an MP sequence with certain noise can be obtained [37].

The magnitude of the noise can have a certain impact on the modeling and correction of an MP. As shown in Equation (2), the extraction of MPs at different frequencies only uses the carrier phase observations of two frequencies. However, with the increase in the frequencies broadcasted by various navigation systems, introducing additional frequency carrier phase observations should be able to obtain MP sequences with less noise. For BDS-3 MEO and IGSO satellites, five signal frequencies can now be broadcasted. If five-frequency carrier phase observations are used to extract an MP at a certain frequency, it can be represented as:

(3) $${\mathrm{MP}}_{j}=\eta_{0}P_{j}+\eta_{j}\Phi_{j}+\sum_{i=1,i\neq k}^{5}\eta_{i}\Phi_{i}$$

where $\eta_{0}$ and $\eta_{j}$ represent the combination coefficients of the pseudorange and carrier phase observations at frequency *j*, respectively, while $\eta_{i}$ denotes the combination coefficients of the carrier phase observations at other frequencies.

From Equation (1), it follows that, for the MP_1_ extraction using Equation (3), when only eliminating the influence of *I*_1,1_, $\eta_{0}\sim \eta_{5}$ should satisfy:

(4) $$\left\{ \begin{array}{l} \eta_{0}=1\\ \eta_{1}+\eta_{2}+\eta_{3}+\eta_{4}+\eta_{5}=-1\\ \eta_{0}k_{1}-\eta_{1}k_{1}-\eta_{2}k_{2}+\eta_{3}k_{3}-\eta_{4}k_{4}-\eta_{5}k_{5}=0\\ \eta_{0}^{2}+\eta_{1}^{2}+\eta_{2}^{2}+\eta_{3}^{2}+\eta_{4}^{2}+\eta_{5}^{2}=\min \end{array}\right.$$

When the effects of *I*_1,1_ and *I*_1,2_ are considered simultaneously, Equation (4) can be combined with Equation (5).

(5) $$2\eta_{0}\nu_{1}-\eta_{1}\nu_{1}-\eta_{2}\nu_{2}-\eta_{3}\nu_{3}-\eta_{4}\nu_{4}-\eta_{5}\nu_{5}=0$$

According to Equations (4) and (5), $\eta_{1}\sim \eta_{5}$ can be obtained by the Lagrange multiplier method. In fact, the solution of Equation (4) should introduce additional parameters, which increases the computational workload. In this work, starting from the observation Equations of the pseudorange and carrier phase, a method for calculating the multi-frequency MP combination coefficients based on the least squares principle is proposed, directly according to the characteristics of the combined parameters.

Equation (4) shows that the coefficients of the MP combination need to satisfy the condition that the combined geometric distance term and ionospheric delay term are both equal to zero. Therefore, when considering only the geometric distance term, *I*_1,1_, and MP, the observation equations for the pseudorange and carrier phase can be simplified as follows:

(6) $$\underset{Z}{\underset{︸}{\left[\begin{array}{c} P_{1}\\ \Phi_{1}\\ \Phi_{2}\\ \Phi_{3}\\ \Phi_{4}\\ \Phi_{5} \end{array}\right]}}=\underset{H}{\underset{︸}{\left[\begin{array}{ccc} 1 & k_{1} & 1\\ 1 & -k_{1} & 0\\ 1 & -k_{2} & 0\\ 1 & -k_{3} & 0\\ 1 & -k_{4} & 0\\ 1 & -k_{5} & 0 \end{array}\right]}}\underset{X}{\underset{︸}{\left[\begin{array}{c} \tilde{\rho }\\ I_{1,1}\\ {\mathrm{MP}}_{1} \end{array}\right]}}+\underset{\varepsilon }{\underset{︸}{\left[\begin{array}{c} \varepsilon_{P_{1}}\\ \varepsilon_{\Phi_{1}}\\ \varepsilon_{\Phi_{2}}\\ \varepsilon_{\Phi_{3}}\\ \varepsilon_{\Phi_{4}}\\ \varepsilon_{\Phi_{5}} \end{array}\right]}}$$

where $\tilde{\rho }$ denotes the sum of the geometric distances of the all frequency-independent terms from the satellite to the receiver.

Assuming that the covariance matrix of the observation is ***Q***, the least squares solution of the estimated parameters ***X*** can be obtained from Equation (6), which is expressed as:

(7) $$X={\left(H^{T}Q^{-1}H\right)}^{-1}H^{T}Q^{-1}Z$$

Equation (7) can also be seen as a combination of the pseudorange observations and carrier phase observations at different frequencies to extract $\tilde{\rho }$, *I*_1,1_, and MP_1_, where the combination coefficients are:

(8) $$x={\left(H^{T}Q^{-1}H\right)}^{-1}H^{T}Q^{-1}$$

In order to satisfy the condition of Equation (4), the transformation matrix $R={\left[\begin{array}{ccc} R_{1} & R_{2} & R_{3} \end{array}\right]}^{T}$ can be defined, where: $R_{1}=R_{2}=\left[\begin{array}{ccc} 0 & 0 & 0 \end{array}\right]$, $R_{3}=\left[\begin{array}{ccc} 0 & 0 & 1 \end{array}\right]$. At this point, the combined $\tilde{\rho }$ and *I*_1,1_ can be kept at 0, while the coefficient of MP_1_ remains at 1, that is:

(9) $$\hat{x}=Rx=R{\left(H^{T}Q^{-1}H\right)}^{-1}H^{T}Q^{-1}$$

According to Equation (9), the combination coefficient of MP_1_ can be expressed as:

(10) $$\eta =\left[\begin{array}{rrrrrr} \eta_{0} & \eta_{1} & \eta_{2} & \eta_{3} & \eta_{4} & \eta_{5} \end{array}\right]=R_{3}{\left(H^{T}Q^{-1}H\right)}^{-1}H^{T}Q^{-1}$$

The optimal coefficient of the MP combination for a certain frequency can be obtained through Equation (10). Assuming that the carrier phase observations of *n* frequencies are used, the MP combination coefficient can be expressed as:

(11) $$\left\{ \begin{array}{l} \eta_{0}=1\\ \eta_{1}=-(\sum_{i=2}^{n}k_{i}{}^{2}-n+1)/C_{n}\\ \eta_{e}=-(\sum_{i=2}^{n}\left(k_{i}{}^{2}-k_{i}k_{e}+k_{i}\right)-(n+1)k_{e}+2)/C_{n}\begin{array}{ccc} & & \left(e=2,3,\cdots ,n\right) \end{array} \end{array}\right.$$

(12) $$C_{n}=\sum_{i=2}^{n-1}\sum_{j=i+1}^{n}{\left(k_{i}^{}-k_{j}^{}\right)}^{2}+\sum_{k=2}^{n}{\left(k_{k}^{}-1\right)}^{2}$$

Equations (11) and (12) provide analytical expressions for the optimal coefficients of multi-frequency MP combinations. When extracting MPs using different frequency observations, it is only necessary to directly bring in the *k_i_* corresponding to different frequencies. However, it is important to note that *k_i_* is not fixed and constant. Although the same carrier phase observations are used, when the frequency of extracting the MP is different, *k_i_* also needs to be updated again.

If the influence of *I*_1,2_ needs to be eliminated when extracting an MP, Equation (6) can be rephrased as:

(13) $$\underset{Z}{\underset{︸}{\left[\begin{array}{c} \begin{array}{c} P_{1}\\ \Phi_{1} \end{array}\\ \Phi_{2}\\ \Phi_{3}\\ \Phi_{4}\\ \Phi_{5} \end{array}\right]}}=\underset{H}{\underset{︸}{\left[\begin{array}{rrrr} 1 & k_{1} & 2\nu_{1} & 1\\ 1 & -k_{1} & -\nu_{1} & 0\\ 1 & -k_{2} & -\nu_{2} & 0\\ 1 & -k_{3} & -\nu_{3} & 0\\ 1 & -k_{4} & -\nu_{4} & 0\\ 1 & -k_{5} & -\nu_{5} & 0 \end{array}\right]}}\underset{X}{\underset{︸}{\left[\begin{array}{l} \tilde{\rho }\\ I_{1,1}\\ I_{1,2}\\ {\mathrm{MP}}_{1} \end{array}\right]}}+\underset{\varepsilon }{\underset{︸}{\left[\begin{array}{c} \begin{array}{l} \varepsilon_{P_{1}}\\ \varepsilon_{\Phi_{1}}\\ \varepsilon_{\Phi_{2}}\\ \varepsilon_{\Phi_{3}}\\ \varepsilon_{\Phi_{4}} \end{array}\\ \varepsilon_{\Phi_{5}} \end{array}\right]}}$$

By updating the transformation matrix ***R***($R_{4}=\left[\begin{array}{ccc} 0 & 0 & \begin{array}{cc} 0 & 1 \end{array} \end{array}\right]$) accordingly, the MP combination coefficients eliminating *I*_1,2_ can be quickly obtained. Taking triple-frequency observations as an example, the combination coefficient when simultaneously eliminating *I*_1,1_ and *I*_1,2_ can be expressed as:

(14) $$\left\{ \begin{array}{l} \eta_{0}=1\\ \eta_{1}=(-2(k_{2}-k_{3})+(\nu_{2}-\nu_{3})-(k_{2}\nu_{3}-k_{3}\nu_{2}))/D_{3}\\ \eta_{2}=(-3k_{3}+2\nu_{3}+1)/D_{3}\\ \eta_{3}=(3k_{2}-2\nu_{2}-1)/D_{3} \end{array}\right.$$

(15) $$D_{3}=-(k_{2}-k_{3})+(\nu_{2}-\nu_{3})+(k_{2}\nu_{3}-k_{3}\nu_{2})$$

**Figure 1:**
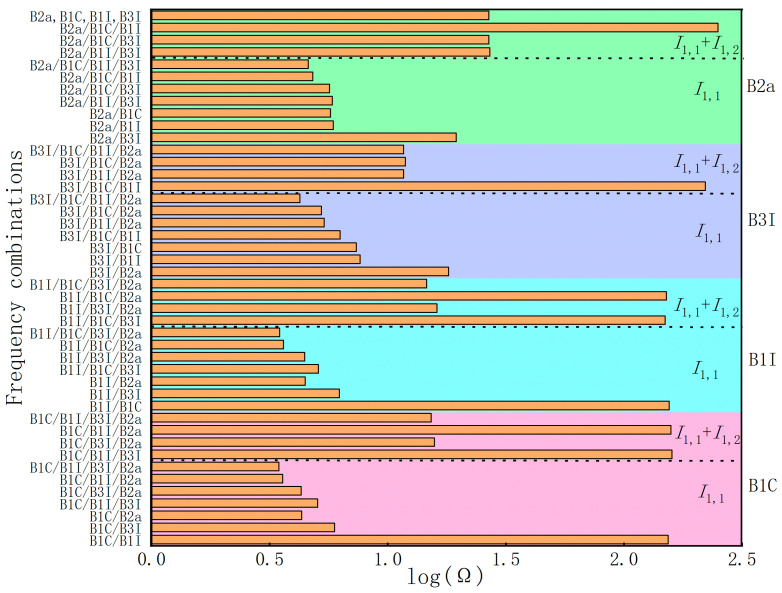
Ω of different frequency combinations for MP extracting.

Taking the B1I frequency as an example, Table 1 shows the combination coefficients and carrier phase observation noise amplification factors ($\Omega =\sqrt{\eta_{1}{}^{2}+\eta_{2}{}^{2}+\cdots +\eta_{n}{}^{2}}$) for extracting MPs using different frequency combinations. The DF, TF, QF, and FF represent dual-frequency, triple-frequency, quad-frequency, and five-frequency, respectively. Figure 1 shows the combination noise of the B1C/B1I/B3I/B2a frequency when extracting MPs through different frequency combinations.

From Table 1 and Figure 1, it can be seen that the Ω varies significantly under different frequency combinations. For example, the Ω of B1I/B1C can be 25–35 times that of other dual-frequency combinations and about 45 times that of quad- and five-frequency combinations. Related studies have pointed out that, when the Ω is large, it will amplify the observation error implied by the carrier phase observations [20,34]. Therefore, combinations of Ω similar to B1I/B1C should be careful not to be used for extracting MPs. The commonly used B1I/B3I combination has a combined noise of 6.245. From Figure 1, it can be seen that the Ω is greater than most combinations. In theory, when the Ω is smaller, the extracted MP sequence contains less noise. Therefore, combinations such as triple-frequency or quad-frequency should be able to extract MP sequences with smaller error fluctuations.

Additionally, when both the *I*_1,1_ and *I*_1,2_ are eliminated simultaneously, the Ω significantly increases and the optimal combination is different from when only *I*_1,1_ is considered. For example, the combination of B1I/B1C/B2a for B1I is relatively optimal for the triple-frequency combination. However, when both *I*_1,1_ and *I*_1,2_ are simultaneously considered, this combination actually has the largest combination noise. Through the analysis of the coefficients of the four frequency combinations, it is found that the contribution of the B3I frequency to the combination is relatively small when considering only *I*_1,1_, while it has the largest contribution when eliminating both *I*_1,1_ and *I*_1,2_ simultaneously. Another point to note is that, when extracting MPs with the same frequency of different frequencies, the combination coefficients are different. Although the expression of the coefficients is the same, due to the change in the first frequency, the corresponding *k*_2_ and *k*_3_ will also change. For instance, when using the combination of B1C/B1I/B2a to extract the MP of each frequency, the corresponding combination noises are $\Omega_{B1C}=3.593$, $\Omega_{B1I}=3.621$, and $\Omega_{B2a}=4.820$, respectively.

## 3. Experiments and Analyses

**Figure 2:**
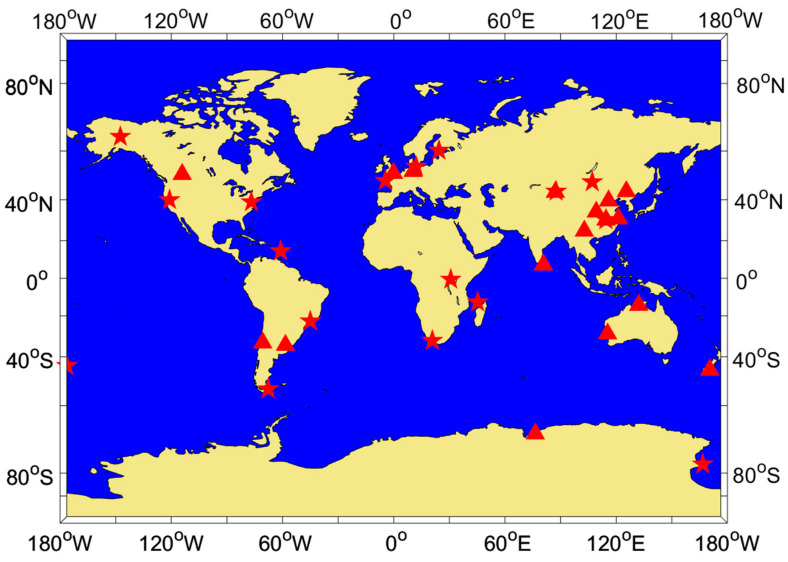
Distribution of experimental stations (Pentagram: MGEX station, triangle: IGMAS station).

In this section, the correctness of the theories proposed in this work and the characteristics of the MPs for each frequency of BDS-3 will be discussed through experiments. The observations of 35 stations (18 MGEX stations and 17 iGMAS stations) over 7 days (DOY 1–7, 2021) were selected, and all stations could receive observations of the B1I, B1C, B3I, and B2a frequencies of BDS-3. The sampling interval for the observations at each station was 30 s. Figure 2 shows the distribution of these stations. Additionally, a set of dynamic vehicle observations (CAR1 station) was also selected to further validate the theories proposed in this work. The data acquisition scheme can be found in reference [38]. Standard deviation (STD), mean, and range were selected as statistics to measure the accuracy.

### 3.1. Comparison of Characteristics of MP Extracted with Different Frequency Combinations

In order to compare the differences in the extracted MP sequences when different frequency combinations were used, taking the B1I and B1C frequencies as examples, four modes with varying combination noise from dual-frequency to quad-frequency were selected to extract the MPs for each frequency. The specific combination frequencies and coefficients are shown in Table 2. Although there were differences in the combined noise of the four modes, each mode listed was the optimal combination coefficient calculated for the corresponding frequency. This section mainly verifies the difference between the different frequency combinations on the obtained MP sequences, without considering the difference between the optimal combination and non-optimal combination among each frequency. In fact, the experimental results can still reflect the difference between them, as the combination noise of the non-optimal combinations also increased correspondingly, and the comparison results should be similar to the results.

**Figure 3:**
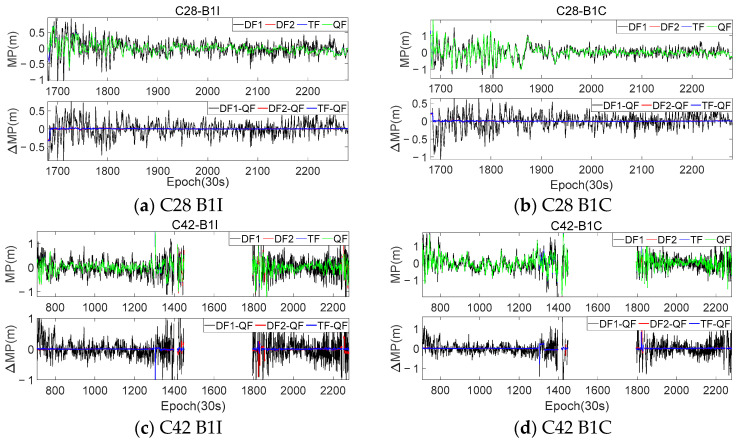
Comparison of MP sequences extracted by different modes of KUN1 station.

Taking the observation of the KUN1 station as an example, Figure 3 presents the MP sequences extracted from the C28 and C40 satellites using different frequencies. In each sub-figure, the upper portion represents the extracted MP sequences for the B1I and B1C frequencies, while the lower portion shows the first-order difference between each mode and the QF mode. Table 3 provides the accuracy statistics for the MP sequences extracted using different combination modes, as well as the improvement relative to DF1.

**Figure 4:**
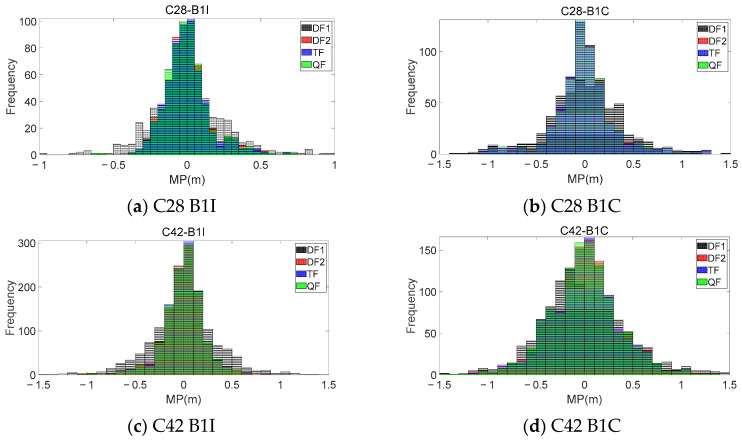
Frequency distribution of MP sequences extracted by different modes of KUN1 stations.

By comparing the four modes, it can be observed that the MP sequences extracted using the triple-frequency and quad-frequency combinations exhibited a similar trend to those extracted using the traditional dual-frequency method, but with smaller fluctuations. This indicates that the coefficient calculated using Equation (11) was correct and optimal. The results also show that if the Ω was small, the fluctuation of the extracted MP sequence was smaller. By comparing this with the QF mode, it can be observed that, when the difference of the Ω was relatively small, the first-order difference between the two sequences generally fluctuated within 3 cm. However, the difference between the QF mode and the DF1 mode still fluctuated at the meter level. This indicates that, when the Ω was small, the extracted sequences were essentially consistent with each other. Additionally, Table 3 also shows that the improvement in the accuracy, relative to the DF1 mode, was consistent across the different combination modes. Therefore, when certain frequency observations were missing, other combination modes could be adaptively selected to extract the MP. Figure 4 presents histograms of the frequency distributions for the MP sequences extracted using different modes. It can be seen that the sequences from the DF2, TF, and QF modes are relatively more concentrated around zero. However, due to the favorable observation environment at the KUN1 station, the representation in the histograms of the frequency distribution is not significant.

**Figure 5:**
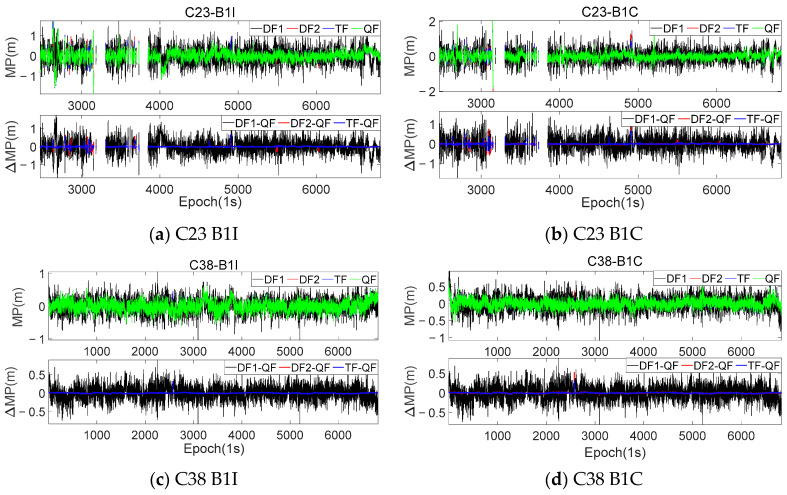
Comparison of MP sequences extracted by CAR1 station with different modes.

**Figure 6:**
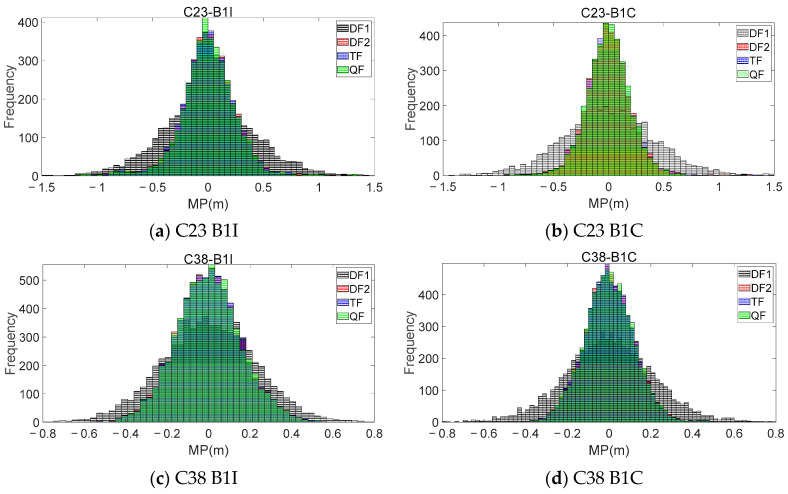
Frequency distribution of MP sequences extracted by different modes of CAR1 stations.

To further validate the theories proposed in this article and compare the characteristics of the MP sequences extracted from the different modes, the same frequency combinations and coefficients shown in Table 2 were used to process the dynamic observations. Similar to Figure 3, Figure 5 presents the results and difference sequences for satellites C23 and C38, while Figure 6 displays histograms of the frequency distribution for the MP sequences extracted using the different modes. Table 4 provides the accuracy statistics for the two satellites.

It can be seen that, due to the significant influence of the surrounding environment during the collection of the dynamic observations, the selected multi-frequency combinations in this work could effectively reduce the fluctuation level and magnitude of the MP sequences. Figure 5 provides a more intuitive representation of the numerical variation range of the MPs extracted using different combinations compared to the DF1 mode. Although the improvement in the accuracy achieved using the DF2, TF, and QF modes for the dynamic observations was not significantly different from that of the DF1 mode, this accuracy improvement was more pronounced compared to the KUN1 station. The B1I and B1C frequencies of the two satellites at the KUN1 station showed accuracy improvements ranging from 38–42% to 13–15%, respectively, while the CAR1 station exhibited even higher improvements, reaching over 30–39% for the B1I frequency and 43–50% for the B1C frequency. The comparison between the two stations highlights the increased importance of using optimal multi-frequency combinations in dynamic experiments.

### 3.2. Analysis of the Influence of Second-Order Ionospheric Delay

In Section 3.1, the influence of the four different frequency combination modes on the MP extraction was compared using the B1I and B1C frequencies as examples. However, the coefficients used were the optimal solutions only when *I*_1,1_ was eliminated, and the influence of the simultaneous elimination of *I*_1,2_ on the extracted MPs was not considered. From the derivation process of the optimal coefficients in Section 2, it can be found that, when only *I*_1,1_ was considered, the dual-frequency combination could only have a unique solution, while the triple-frequency, quad-frequency, and five-frequency combinations could obtain the optimal solution with the minimum combination noise due to the presence of redundant observations. Similarly, when considering both *I*_1,1_ and *I*_1,2_, Equations (4) and (5) need at least three frequency observations to achieve a unique solution, and the quad-frequency and five-frequency combinations could obtain the optimal solution. Based on the findings in Section 3.1, when the combination noise was similar, the extracted MP sequences were also very similar. In this section, a further analysis is conducted to examine the impact of ionospheric delay on these MP sequences. Taking the triple-frequency and quad-frequency combinations as examples, the influence of second-order ionospheric delay on the MP sequences extracted using different frequency combinations is analyzed. Table 5 provides the selected combination modes and their corresponding coefficients for the B1C, B3I, and B2a frequencies. The combination modes for the B1I frequency can be found in Table 1, where B1I/B1C/B3I, B1I/B1C/B2a, B1I/B3I/B2a, and B1I/B1C/B3I/B2a are represented as B1ITF1, B1ITF2, B1ITF3, and B1IQF1, respectively.

**Figure 7:**
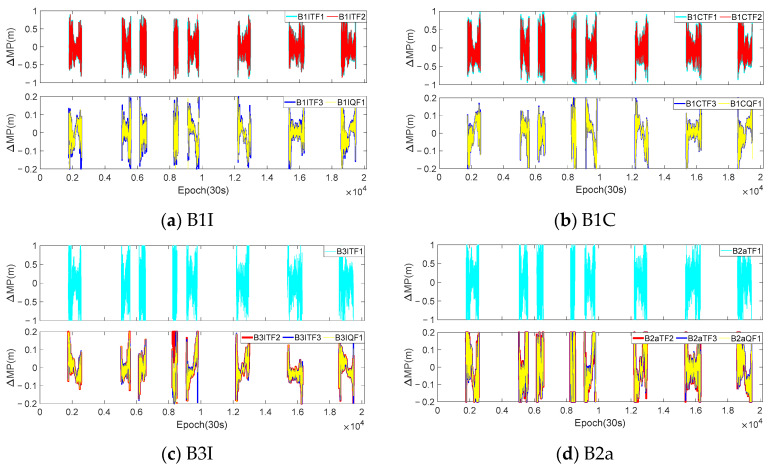
The differenced MP sequences of C28 satellite at WUH2 station under each mode.

**Figure 8:**
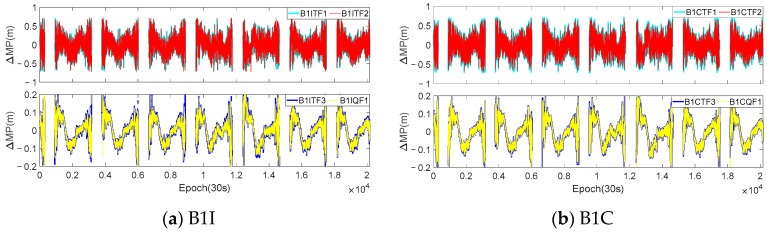

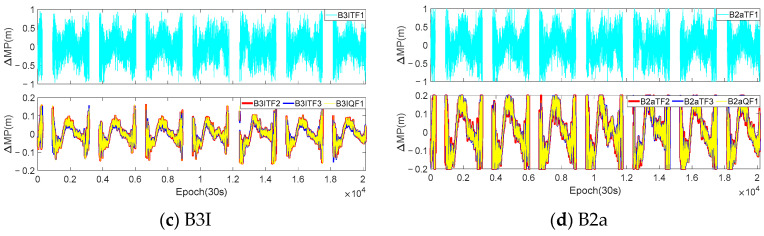
The differenced MP sequences of C38 satellite at WUH2 station under each mode.

Theoretically, regardless of whether the *I*_1,2_ was eliminated or not, the coefficients of the pseudorange observations involved in the combination were all 1, which meant that the truth values contained in the extracted sequences were consistent. However, due to the different combination coefficients of the two methods, the errors contained in the extracted sequence also differed. In order to analyze the impact of eliminating *I*_1,2_ on the extraction of the MP sequences, the optimal coefficients of the same mode in Table 5 were used to extract the sequences for eliminating *I*_1,1_ and *I*_1,1_/*I*_1,2_, and a difference was made between the two sets of sequences. The difference between the two sets of sequences could be considered as the influence of *I*_1,2_ under that mode. Taking the continuous 7-day observations from the WUH2 station as an example, Figure 7 and Figure 8, respectively, show the difference sequences for C28 (MEO satellite) and C38 (IGSO satellite) at various frequencies. B1CTF1 in the figures represents the first-order difference sequence for the B1CTF1 mode, and the meanings of the other sequences are similar. Table 6 provides statistical information on the accuracy of all the BDS-3 MEO and IGSO satellites at the experimental stations for the 7 days under each mode.

Based on Figure 7 and Figure 8, as well as Table 6, it can be observed that:

(1) There was a clear correlation between the magnitude of the fluctuations in the difference sequences and the ΔΩ. The ΔΩ for the B1ITF1, B1ITF2, B1CTF1, B1CTF2, B3ITF1, and B2aTF1 modes were all above 140. The STD and range values for each sequence were also greater than 0.24 m and 1.5 m, respectively, which were 3–5 times larger than those for the other modes. Among them, the B2aTF1 mode had the largest ΔΩ, which was 245.626. Its STD and range were 0.401 m and 2.478 m, respectively, which were also significantly higher than those of the other modes. With the exception of the MP extraction mode for the B2a frequency, the fluctuation range of the differenced sequences for other modes was less than 0.5 m and the STD was basically around 0.05–0.07 m.

(2) According to reference [32,33], when only *I*_1,1_ is considered, the anti-multipath performance of the B2a frequency is better than that of other BDS-3 frequencies. However, the experiments showed that, when eliminating *I*_1,2_ simultaneously, the MPs of the B2a frequency extracted using different modes exhibited larger fluctuations. Table 6 also indicates that, except for modes such as B2aTF1 with significant noise, the STDs for the other three modes were approximately 2–3 times larger than those for the other frequency modes. From the statistical results, it can be seen that the averages of the STDs and ranges of the B2aTF2, B2aTF3, and B2aQF1 modes were 0.113 m and 0.726 m, respectively, which were about 1.8, 1.7, and 2.2 times and 1.6, 1.7, and 1.9 times greater than those of the B1I, B1C, and B3I frequency combinations. The anti-multipath performance of the B3I frequency was also relatively superior, and the combination with a smaller ΔΩ corresponded to a lower STD and range compared to those of the other frequencies. This characteristic was contrary to that of the B2a frequency, and further research is needed in the future.

(3) By comparing the differenced MP sequences of the C28 and C38 satellites at various frequencies, it could be found that, regardless of the magnitude of the fluctuations in the differenced sequences, the differenced sequences of the C38 satellite at each frequency exhibited a clear periodicity. However, the periodicity of the C28 satellite was not prominent. Compared with other IGSO and MEO satellites, they all showed the same periodicity pattern. Additionally, when comparing the four frequencies, it could be observed that their periodic patterns were consistent.

**Figure 9:**
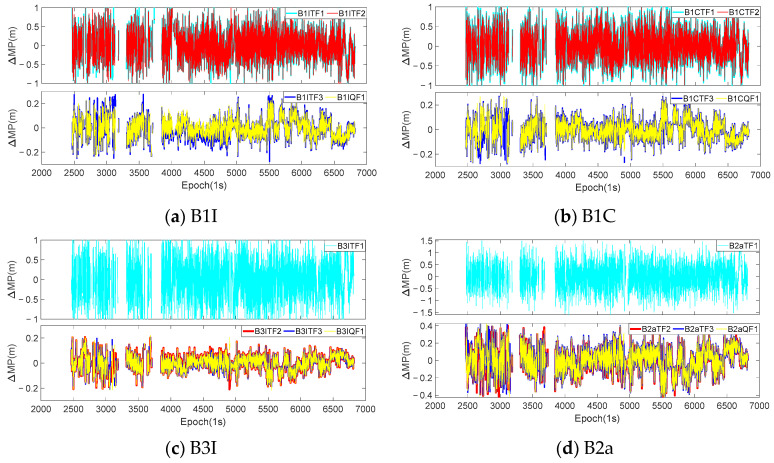
The differenced MP sequences of C23 satellite at CAR1 station under each mode.

**Figure 10:**
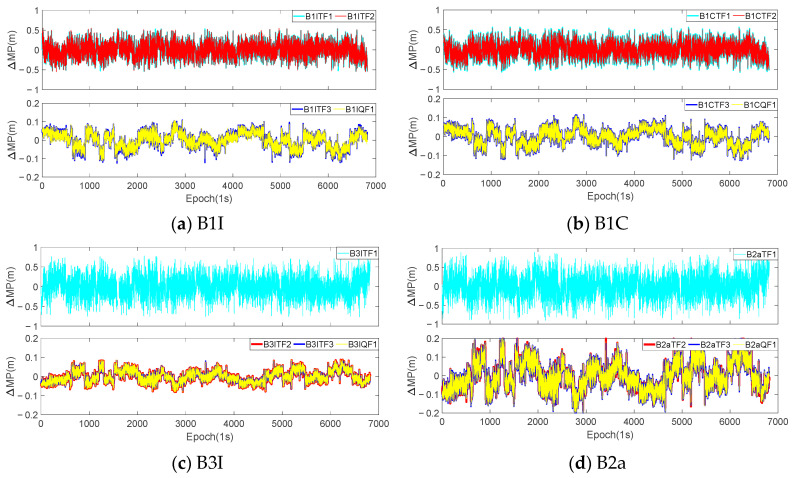
The differenced MP sequences of C38 satellite at CAR1 station under each mode.

In this section, the analysis of the MP difference sequences under the different modes was also conducted using the dynamic observations from Section 3.1. Figure 9 and Figure 10 provide the MP difference sequences extracted with the consideration of *I*_1,2_ for various frequencies of the C23 and C38 satellites at the CAR1 station. Table 7 presents the accuracy statistical information of all the MEO and IGSO satellites at the CAR1 station using different modes.

According to the experimental results, it can be concluded that the fluctuation magnitude of the differenced MP sequences of the BDS-3 IGSO satellites at the CAR1 station was smaller than that of the MEO satellites. The STD and range for each frequency were approximately half of those for the MEO satellites. In addition, similar to the WUH2 station, the fluctuation amplitude of each sequence was positively correlated with the ΔΩ. The fluctuation magnitude of the differenced sequences for the B2a frequency in various modes remained higher than that of the B1I, B1C, and B3I frequencies and the STD and range were also around 1.5–2.0 times larger. The accuracy of the B3I frequency was also better than that of the other frequencies. Due to the observation time being only 2 h, all the sequences fluctuated around the zero value, and the mean values were in the millimeter or sub-millimeter level. The IGSO satellites did not exhibit periodicity. Compared with the WUH2 station, although the observation at the CAR1 station was more affected by the environment, overall, the magnitude of the second-order ionospheric delay remained consistent. For modes with a high ΔΩ, the STD and range of the CAR1 station were slightly higher than those of the WUH2 station. However, when the ΔΩ was small, the STD and range were actually better than that of the WUH2 station.

### 3.3. Analysis of the Correlation between MP and Elevation of BDS-3 Satellite

Based on the previous experiments, it is known that the impact of the BDS-3 satellite’s MP on the pseudorange observations can reach the order of decimeters or even meters, and it needs to be corrected during precise positioning. Currently, there have been numerous studies discussing MP correction methods for BDS-2 satellites, but relatively fewer studies have been conducted for BDS-3 satellites [30,39]. Referring to the relevant research on BDS-2, this section focuses on analyzing the relationship between the MPs and satellite elevations for BDS-3 MEO and IGSO satellites. It should be noted that this analysis only examined their correlation and did not investigate the modeling algorithms, as that would require other processing methods, which will be addressed in future research. For the analysis of this correlation, the MP sequences extracted using the QF mode in Section 3.1 were utilized. Furthermore, in order to avoid MPs being applied to specific satellites, uniform processing was applied to a specific type of satellite at each station to reflect its statistical characteristics. As of February 2023, the distribution of BDS-3 MEO and IGSO satellites can be found in Table 8. The satellites marked with bold numbers indicate their current status as on-orbit testing and do not participate in statistics.

Taking IGSO satellites as an example, assuming that a station observes a total of *n* IGSO satellites during a certain period and that the effective dataset of the *f_i_* frequency for each satellite is $X_{f_{i}}^{n}=\{X_{1},X_{2},\cdots ,X_{n-1},X_{n}\}$, the dataset composed of the satellite elevation (*ele*) and MP for all satellites and all epochs is $Q_{BDS-2}^{IGSO}=\left\{\begin{array}{cc} ele(X_{f_{i}}^{n}) & MP(X_{f_{i}}^{n}) \end{array}\right\}$. Due to the large amount of data, the elevation is divided into different ranges with steps of 0.1°, namely: *d*_0_ = 0°, *d*_1_ = 0.1°, *d*_2_ = 0.2°, *d_k_* = (*k*/10)°, …, *d*_900_ = 90°. The dataset of all the epochs in $Q_{BDS-2}^{IGSO}$ is selected with the elevation between *d_k_*_−1_ and *d_k_*, and a new dataset $Q_{BDS-2}^{IGSO}(d_{k})=\left\{\begin{array}{cc} ele(d_{k}) & MP(d_{k}) \end{array}\right\}$ is formed. The average of $MP(d_{k})$ is taken as the MP corresponding to the *f_i_* frequency when the height angle is *d_k_’ = d_k_* − 0.05°.

**Figure 11:**
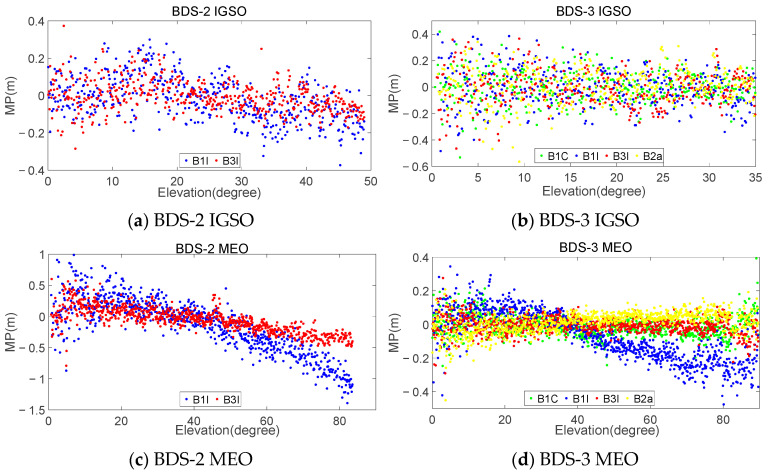
Relationship between satellite elevation and MP of MAYG station.

**Figure 12:**
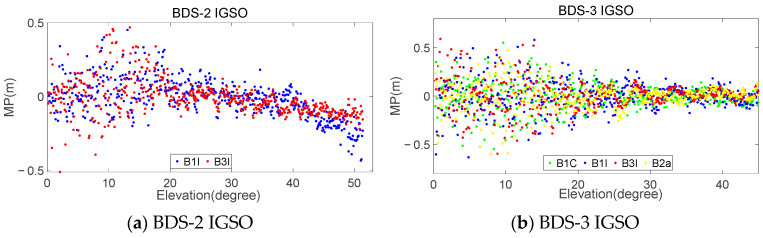

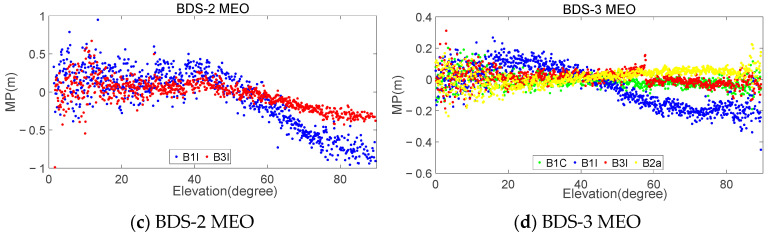
Relationship between satellite elevation and MP of OWMG station.

By using this method, a dataset consisting of elevation and MP can be obtained for each type of satellite and frequency, with both the elevation and MP containing 900 elements. Considering that different stations receive satellites at different elevations and that the maximum and minimum elevation for receiving a particular satellite may vary, there may be cases in the dataset where the MP is empty for certain elevation angles. In such cases, the maximum elevation in $Q(d_{k})$ can be adaptively set based on the specific satellite reception conditions at each station. Figure 11 and Figure 12 provide examples of the statistical results of the MPs and satellite elevations for MAYG and OWMG stations, respectively.

The analysis of the statistical results for all the stations in Figure 2 reveals that the MPs of the MEO and IGSO satellites of BDS-3 exhibited similar systematic biases related to elevation, similar to BDS-2 satellites. Both of them showed more significant changes in MEO satellite systematic biases with elevation. The correlation strength between the MP and elevation varied significantly among different types of satellites and frequencies. The anti-multipath performance of BDS-3 at various frequencies was better than that of BDS-2. The MP of BDS-3 fluctuated around −0.5 m-0.5 m, while BDS-2 mainly fluctuated around −1.0 m-1.0 m. Comparing the different frequencies, it can be seen that the MP fluctuation amplitude of the B2a and B3I frequencies was smaller, while that of the B1C and B1I frequencies was larger. Additionally, the magnitude of the MP fluctuations varied across different satellite orbits for the same frequency. Overall, for BDS-3 satellites, the MP magnitude of the MEO and IGSO satellites was roughly equivalent, while for BDS-2 satellites, the magnitude of the MEO satellites was greater than that of the IGSO.

In order to quantify the correlation between the MP and elevation for each frequency, the correlation coefficient was calculated for different frequencies at each station. The calculation equation is [40]:

(16) $$r_{XY}=\frac{\sum_{i=1}^{n}\left(X-\bar{X})(Y-\bar{Y}\right)}{\sqrt{\sum_{i=1}^{n}{\left(X-\bar{X}\right)}^{2}•\sum_{i=1}^{n}{\left(Y-\bar{Y}\right)}^{2}}}$$

where ***X*** and ***Y*** are the two vectors for the correlation to be determined, $\bar{X}$ and $\bar{Y}$ are the average values corresponding to each vector, and *n* is the length of the vector. The value of *r_xy_* is between −1 and 1, with *r_xy_* > 0 indicating a positive correlation and *r_xy_* < 0 indicating a negative correlation.

Generally, the absolute of *r* indicates the strength of the correlation. Typically, |r|≥0.8 indicates a very strong correlation, 0.6≤|r|< 0.8 indicates a strong correlation, 0.4≤|r|< 0.6 indicates a moderate correlation, 0.2≤|r|< 0.4 indicates a weak correlation, and |r|< 0.2 is uncorrelated [41]. Table 9 shows the average correlation coefficient of each experimental station. From Table 9, it can be observed that, for GEO satellites, the absolute values of the correlation coefficients for each frequency ranged from 0.06 to 0.13, indicating a clear lack of correlation. For IGSO and MEO satellites, the absolute values of the correlation coefficients for each frequency ranged from 0.42 to 0.56 and 0.63 to 0.80, respectively, showing moderate and strong correlations. Additionally, the correlation between the MEO and IGSO satellites of BDS-2 was stronger at the B1I and B3I frequencies than that of BDS-3. Among the four frequencies of BDS-3, the B2a frequency exhibited the strongest correlation.

## 4. Summary and Conclusions

The broadcasting of the multi-frequency observations in various navigation systems presents new opportunities for high-precision PNT services. However, the pseudorange and carrier phase observations of each frequency are subject to various interferences during signal propagation. As one of the errors affecting the pseudorange observations, MPs have also been studied by many scholars. On the basis of existing research, the MP characteristics of BDS-3 satellites at various frequencies were studied based on multi-frequency observations in this work. Through the analysis of static and dynamic observations, the main conclusions can be drawn as follows:

(1) Starting from the pseudorange and carrier phase observation equations, a direct multi-frequency MP combination coefficient calculation method based on the least squares principle was proposed. This method was simple in its calculation and could effectively utilize the observation information of each frequency. On this basis, a detailed formula for calculating the MP combination coefficients for eliminating *I*_1,1_ and *I*_1,2_ was derived, and a unified analytical expression was summarized when only eliminating *I*_1,1_. Compared with the traditional dual-frequency MP combination extraction results, the trend of the MP sequence extracted by the TF and QF combination was basically consistent with it, and the fluctuation amplitude was smaller, which confirmed the correctness of the proposed method.

(2) The accuracy of the MP sequences extracted from the four modes with different Ω was compared by using static and dynamic observations. The results showed that the magnitude of Ω directly affected the fluctuation amplitude of the extracted MP sequences. When there was a significant difference in Ω, the difference in the extracted MP sequences could reach the decimeter level. When the difference of Ω was small, the difference in the extracted MP sequences was generally within 3 cm. Compared to the DF1 mode, the modes with a smaller Ω showed an average improvement of over 25% in the accuracy of the extracted MP sequences, with an even higher accuracy improvement for dynamic observation. Additionally, the selected combinations were the optimal combination coefficients for each frequency combination, which also indirectly reflected the comparison of the accuracy between the optimal and non-optimal combinations in the MP extraction.

(3) Taking the triple-frequency and quad-frequency combinations as examples, the influence of *I*_1,2_ on the extraction of the MPs for the different frequencies and modes was analyzed by taking the first-order difference with the MP sequence obtained by only eliminating *I*_1,1_. The fluctuation amplitude of the differenced MP sequence also showed a clear correlation with ΔΩ, with the STD and range differing by 3–5 times. The B2a and B3I frequencies exhibited better anti-multipath performances. However, under the same level of combination noise, the STD and range of the differenced MP sequence of the B2a frequency were both more than 1.5 times larger than those of the other three frequencies, while the STD and range of the B3I frequency were the smallest.

(4) The impact of *I*_1,2_ on the IGSO and MEO satellites of BDS-3 was different. In different modes, the first-order difference sequences of the four frequencies of the IGSO satellites always exhibited a significant periodicity, while the periodicity of the MEO satellites was not significant. In addition, for the static observation, the magnitude of the differenced MP sequences between the two types of satellites was basically consistent, but for the dynamically collected observation, the STD and range of the IGSO satellites were about half those of the MEO satellites.

(5) Based on the observations from all the experimental stations, the correlation between the MPs of the IGSO and MEO satellites and the satellite elevation was analyzed. The MP of the MEO satellites showed a strong correlation with the elevation, while the correlation for the IGSO satellites was of moderate strength. The correlation between the MEO and IGSO satellites of BDS-2 at the B1I and B3I frequencies was stronger than that of BDS-3. Among the four frequencies of BDS-3, the B2a frequency exhibited the strongest correlation. Overall, the anti-multipath performance of BDS-3 frequencies was superior to that of BDS-2.

This work focused on the method of extracting MPs using multi-frequency observations and analyzed the characteristics of MPs under different scenarios. However, in practical positioning processes, it is necessary to consider how to correct such errors to improve the positioning accuracy. This involves addressing issues such as the extraction of the true information from MPs and the construction of high-precision correction models. These aspects are important considerations for multi-frequency and multi-system precise positioning and will be one of the future research directions.

## Figures and Tables

**Table 1:** MP combination coefficients of B1I frequency.

Frequency Number	*P*	Φ					Ω
	B1I	B1I	B1C	B3I	B2b	B2a	
Eliminate *I*_1,1_							
DF	1.000	109.502	−110.502	/	/	/	155.568
	1.000	−4.887	/	3.887	/	/	6.245
	1.000	−3.974	/	/	2.974	/	4.964
	1.000	−3.629	/	/	/	2.629	4.481
TF	1.000	−2.292	−2.507	3.799	/	/	5.096
	1.000	−1.741	−1.844	/	/	2.585	3.621
	1.000	−3.754	/	0.386	/	2.368	4.455
QF	1.000	−1.871	−1.964	0.782	/	2.053	3.491
FF	1.000	−1.870	−1.948	0.356	1.040	1.422	3.244
Simultaneously eliminate *I*_1,1_ and *I*_1,2_							
TF	1.000	105.310	−106.452	0.142	/	/	149.741
	1.000	106.580	−107.647	/	/	0.068	151.483
	1.000	−7.784	/	12.834	/	−6.050	16.183
QF	1.000	−2.763	−4.726	12.270	/	−5.781	14.627
FF	1.000	−2.755	−4.684	11.546	1.583	−6.689	14.494

**Table 2:** MP combination of B1C and B1I frequencies selected in the experiment.

Combination Mode	Combination Frequencies	Combination Coefficients	Ω
B1I			
DF1	(B1I, B1C)	(1, 109.502, −110.502)	155.568
DF2	(B1I, B3I)	(1, −4.887, 3.887)	6.245
TF	(B1I, B1C, B3I)	(1, −2.292, −2.507, 3.799)	5.096
QF	(B1I, B1C, B3I, B2a)	(1, −1.871, −1.964, 0.782, 2.053)	3.491
B1C			
DF1	(B1C, B1I)	(1, −109.502, 108.502)	154.154
DF2	(B1C, B3I)	(1, −4.687, 3.687)	5.964
TF	(B1C, B1I, B3I)	(1, −2.489, −2.276, 3.765)	5.054
QF	(B1C, B1I, B3I, B2a)	(1, −1.951, −1.858, 0.774, 2.035)	3.464

**Table 3:** Accuracy statistics of MP sequences extracted by different modes of KUN1 stations.

	PRN	DF1	DF2	TF	QF
B1I					
STD/m	C28	0.270	0.161/40.3%	0.160/40.3%	0.157/41.8%
	C42	0.377	0.231/38.7%	0.228/39.5%	0.223/40.8%
mean/m	C28	−7.744 × 10^−16^	8.808 × 10^−16^	1.182 × 10^−15^	−1.744 × 10^−16^
	C42	−1.145 × 10^−14^	−1.001 × 10^−15^	−1.695 × 10^−15^	2.270 × 10^−15^
B1C					
STD/m	C28	0.396	0.342/13.6%	0.341/13.9%	0.341/13.9%
	C42	0.463	0.393/15.1%	0.395/14.7%	0.393/15.1%
mean/m	C28	3.577 × 10^−16^	−1.773 × 10^−16^	1.750 × 10^−15^	1.833 × 10^−16^
	C42	4.006 × 10^−15^	−3.303 × 10^−16^	2.608 × 10^−16^	−1.716 × 10^−15^

**Table 4:** Accuracy statistics of MP sequences extracted by different modes of CAR1 stations.

	PRN	DF1	DF2	TF	QF
B1I					
STD/m	C23	0.431	0.270/37.4%	0.272/36.9%	0.263/39.0%
	C38	0.227	0.159/30.0%	0.158/30.4%	0.158/30.4%
mean/m	C23	−2.80 × 10^−15^	−5.78 × 10^−16^	−1.54 × 10^−16^	1.56 × 10^−16^
	C38	2.67 × 10^−14^	6.48 × 10^−16^	4.70 × 10^−18^	8.41 × 10^−16^
B1C					
STD/m	C23	0.425	0.228/46.4%	0.220/48.2%	0.213/49.9%
	C38	0.215	0.121/43.7%	0.121/43.7%	0.120/44.0%
mean/m	C23	2.43 × 10^−15^	1.04 × 10^−15^	−6.68 × 10^−16^	−3.39 × 10^−16^
	C33	8.28 × 10^−15^	1.30 × 10^−17^	−1.22 × 10^−16^	−6.51 × 10^−16^

**Table 5:** Combination modes of simultaneous eliminate *I*_1,1_ and *I*_1,2_ for B1C, B3I, and B2a frequencies.

Mode	Combination Frequencies	Combination Coefficients		$\Omega_{I_{1,1}}$	$\Omega_{I_{1,2}}$	ΔΩ
		*I*_1,1_	*I*_1,1_ + *I*_1,2_			
B1CTF1	B1C, B1I, B3I	1, −2.489, −2.276, 3.765	1, −113.491, 112.631, −0.140	5.054	159.894	154.84
B1CTF2	B1C, B1I, B2a	1, −1.832, −1.729, 2.561	1, −112.314, 111.381, −0.067	3.593	158.178	154.585
B1CTF3	B1C, B3I, B2a	1, −3.644, 0.389, 2.255	1, −7.474, 12.499, −6.025	4.303	15.760	11.457
B1CQF1	B1C, B1I, B3I, B2a	1, −1.951, −1.858, 0.774, 2.035	1, −4.845, −2.793, 12.812, −6.174	3.464	15.282	11.818
B3ITF1	B3I, B1C, B1I	1, 4.781, −3.023, −2.758	1, 10.181, 150.478, −161.659	6.293	221.090	214.797
B3ITF2	B3I, B1C, B2a	1, 0.550, −4.379, 2.829	1, −7.960, −1.688, 8.648	5.242	11.874	6.632
B3ITF3	B3I, B1I, B2a	1,0.538,−4.479,2.941	1, −7.759, −1.793, 8.552	5.385	11.685	6.3
B3IQF1	B3I, B1C, B1I, B2a	1,1.012,−2.346,−2.231,2.565	1, −7.786, −0.230, −1.549, 8.565	4.252	11.680	7.428
B2aTF1	B2a, B1C, B1I	1, 3.573, −2.355, −2.218	1, 7.734, 172.586, −181.320	4.820	250.446	245.626
B2aTF2	B2a, B1I, B3I	1, 3.215, −4.827, 0.612	1, 17.543, 2.033, −20.576	5.832	27.115	21.283
B2aTF3	B2a, B1C, B3I	1, 3.094, −4.718, 0.624	1, 17.434, 1.913, −20.347	5.677	26.863	21.186
B2aQF1	B2a, B1C, B1I, B3I	1, 2.810, −2.528, −2.404, 1.212	1, 17.394, 2.614, −0.744, −20.264	4.618	26.843	22.225

**Table 6:** Accuracy statistics of MP difference sequences in different modes of experimental stations (unit: m).

Mode	Mean	STD	Range	Mode	Mean	STD	Range
B1ITF1	−0.006	0.247	1.599	B1CTF1	−0.013	0.268	1.689
B1ITF2	−0.006	0.252	1.633	B1CTF2	−0.012	0.255	1.605
B1ITF3	−0.001	0.063	0.451	B1CTF3	0.002	0.067	0.441
B1IQF1	0.000	0.059	0.441	B1CQF1	0.001	0.065	0.429
B3ITF1	−0.008	0.366	2.275	B2aTF1	0.003	0.401	2.478
B3ITF2	−0.001	0.052	0.389	B2aTF2	−0.003	0.113	0.724
B3ITF3	−0.005	0.049	0.367	B2aTF3	−0.003	0.112	0.713
B3IQF1	−0.005	0.051	0.382	B2aQF1	−0.004	0.114	0.740

**Table 7:** Accuracy statistics of MP sequences in different modes of CAR1 station (unit: m).

Mode	Mean	STD	Range	Mode	Mean	STD	Range
B1ITF1	−0.002	0.275	1.665	B1CTF1	0.000	0.288	1.712
B1ITF2	−0.003	0.280	1.683	B1CTF2	0.000	0.274	1.626
B1ITF3	−0.003	0.060	0.393	B1CTF3	−0.001	0.061	0.396
B1IQF1	0.000	0.056	0.359	B1CQF1	0.000	0.060	0.389
B3ITF1	0.001	0.381	2.274	B2aTF1	0.003	0.415	2.466
B3ITF2	0.000	0.044	0.294	B2aTF2	−0.001	0.100	0.610
B3ITF3	−0.001	0.041	0.284	B2aTF3	0.001	0.104	0.633
B3IQF1	0.000	0.045	0.307	B2aQF1	0.001	0.105	0.635

**Table 8:** Satellite distribution in each orbit of BDS-3.

	BDS-2 (B1I, B3I,B2I)	BDS-3 (IGSO/MEO: B1C,B1I,B3I,B2a)
IGSO	C06, C07, C08, C09, C10, C13, C16	**C31**, C38, C39, C40, **C56**
MEO	C11, C12, C14	C19, C20, C21, C22, C23, C24, C25, C26, C27, C28, C29, C30, C32, C33, C34, C35, C36, C37, C41, C42, C43, C44, C45, C46, **C57, C58**

**Table 9:** The correlation coefficient between MP and satellite elevation.

	BDS-2		BDS-3			
	B1I	B3I	B1C	B1I	B3I	B2a
GEO	−0.06	−0.13	—	−0.09	−0.10	—
IGSO	−0.56	−0.54	0.42	−0.42	−0.44	0.51
MEO	−0.79	−0.80	0.63	−0.70	0.63	0.78

## Data Availability

Not applicable.
